# Dissemination of EFFORT trial results in Indonesia: a stakeholder meeting report on tafenoquine and primaquine for radical cure of *Plasmodium vivax*

**DOI:** 10.1186/s12936-026-05948-3

**Published:** 2026-05-27

**Authors:** Ayodhia Pitaloka Pasaribu, Vincent Jimanto, Framita Ainur, Novita Rohdearni Saragih, Halik Hadi, Dedi Sahat Parningotan Siagian, Budi Junaraman Sinaga, Ari Winasti Satyagraha, Ferdinand J. Laihad, Ajib Diptyanusa, Reynold Rizal Ubra, Obet Tekege, Lusi Darmayanti, Herdiana Herdiana, Jacklyn Adella, Kylie Mannion, Ella Curry, Jeanne Rini Poespoprodjo, Enny Kenangalem, Faustina Helena Burdam, Inge Sutanto, Erni Juwita Nelwan, Gabriel Jason Ganadhi, Bijaya Shrestha, Hellen Mnjala, William A. Hawley, Hellen Dewi Prameswari, Minerva Theodora, Fadjar Surya Mensing Silalahi, Hermawan Susanto, Febry Immanuella, Riskha Tiara Puspadewi, Sri Budi Fajariyani, Iriani Samad, Dedy Supriyanto, Nurul Muhafilah, Rahmad Isa, Ric N. Price, Kamala Thriemer

**Affiliations:** 1 Medical Faculty, Universitas Sumatera Utara, Medan, Indonesia; 2 Yayasan Penguatan Kesehatan Masyarakat Tridarma, Medan, Indonesia; 3 Provincial Health Office, Medan, North Sumatra Indonesia; 4 District Health Office, Batu Bara Regency, Indonesia; 5 Eijkman Research Center for Molecular Biology, National Research and Innovation Agency, Cibinong, Indonesia; 6 EXEINS Health Initiative, Jakarta, Indonesia; 7 Directorate of Communicable Disease, Ministry of Health, Jakarta, Indonesia; 8 Indonesia Country Office, World Health Organization, Jakarta, Indonesia; 9 District Health Office, Mimika, Central Papua Indonesia; 10 Provincial Health Office, Bandar Lampung, Lampung Indonesia; 11 Global and Tropical Health Division, Menzies School of Health Research, Charles Darwin University, Darwin, Australia; 12 Centre for Child Health-PRO, Faculty of Medicine, Public Health and Nursing, Universitas Gadjah Mada, Yogyakarta, Indonesia; 13 Yayasan Pengembangan Kesehatan dan Masyarakat Papua, Timika, Indonesia; 14 University of Indonesia, Jakarta, Indonesia; 15 Independent Consultant, Jakarta, Indonesia; 16 Directorate of Medical Devices Control, Ministry of Health, Jakarta, Indonesia; 17 Directorate of Vulnerable Group Health Services, Jakarta, Indonesia; 18 Mahidol Oxford Research Unit, Faculty of Tropical Medicine, Mahidol University, Bangkok, Thailand; 19 Center for Tropical Medicine and Global Health, Nuffield Department of Medicine, University of Oxford, Oxford, UK

## Abstract

The EFFORT clinical trial was conducted in four countries (Cambodia, Ethiopia, Indonesia, and Pakistan) to evaluate the safety and effectiveness of high-dose primaquine (7 mg/kg over 7 days) and single-dose tafenoquine (300 mg) compared with the standard 14-day low-dose primaquine regimen for the radical cure of patients with *Plasmodium vivax* malaria. In Indonesia, both regimens were co-administered with dihydroartemisinin–piperaquine. This paper reports on a national stakeholder meeting held in May 2025 to disseminate Indonesia-specific findings and discuss their implications for malaria control and elimination policy. Discussions centred on (i) the evidence gaps regarding tafenoquine when combined with artemisinin combination therapies (ACTs), (ii) the feasibility of implementing high-dose primaquine in Indonesia as a near-term option, and (iii) the need for subnational strategies that reflect the country’s diverse epidemiological context. Stakeholders highlighted the need for further studies on tafenoquine with ACTs, while recognising high-dose primaquine as an immediately implementable option pending strengthened G6PD testing. The discussion emphasised the importance of tailoring strategies to Indonesia’s diverse settings, supported by strong political commitment to elimination.

## Background

Malaria remains an important public health challenge in Indonesia, with almost 543,965 confirmed cases reported in 2024 an increase of 30% from 2023 [1]. In the last two decades the country has made substantial progress towards malaria elimination, however the remaining burden of disease is unevenly distributed. The provinces of Papua and West Papua continue to report high levels of transmission, and together account for more than 93% of all cases nationally, whereas many districts in Java, Bali, and parts of Sumatera have achieved or are nearing malaria elimination [1]. *Plasmodium vivax* is common throughout the country, responsible for an estimated 45% of all malaria cases [1].

Indonesia has committed to malaria elimination by 2030 as outlined in its National Malaria Strategic Plan. In 2026 a target has been set to ensure that 86% of the Indonesian territory is free from malaria transmission [2]. This ambitious goal will require targeted approaches that reflect the diverse transmission settings across the archipelago.

The effective treatment of *P. vivax* malaria requires treatment of both the blood-stage asexual parasites and dormant liver stages (hypnozoites). In Indonesia, the standard regimen is currently a total dose of 3.5 mg/kg primaquine administered over 14 days (0.25 mg/kg/day) [3]. Since routine testing for glucose-6-phosphate dehydrogenase (G6PD) deficiency is not widely available this low dose primaquine regimen is used to minimise the risk of drug-induced haemolysis in individuals with G6PD deficiency, a common enzyme deficiency present in up to 20% of the population in malaria-endemic regions. The effectiveness of this treatment strategy is limited by two challenges. Firstly, adherence to the complete 14-day regimen is often very poor thereby reducing the effectiveness of treatment [4, 5]. In routine practice in Papua, Indonesia, the effectiveness of the 14-day regimen has been estimated to be as low as 12% [5]. Second, in a recent metanalysis the low-dose primaquine regimen (3.5 mg/kg total dose) was shown to have suboptimal anti-relapse efficacy compared to the higher-dose regimen (7 mg/kg total dose), which was estimated to halve the risk of recurrent malaria in most geographic settings [6].

In 2024, the World Health Organization (WHO) updated their antimalarial treatment guidelines to recommend a higher total dose of primaquine (7 mg/kg) administered over 7 or 14 days. A single-dose of tafenoquine (300 mg) was also recommended as an alternative anti-relapse regimen, although the latter is currently restricted to South America [7]. Both high-dose primaquine and tafenoquine require prior G6PD testing to ensure patient safety.

Tafenoquine offers a promising alternative to improve adherence through single-dose administration. However, its use is currently restricted to co-administration with chloroquine. This restriction follows findings from the INSPECTOR trial in Papua, Indonesia, in which patients treated with tafenoquine plus dihydroartemisinin-piperaquine (DHA-piperaquine), the current first-line schizontocidal treatment in Indonesia, had a very high risk of recurrent malaria [8]. These results have been attributed to either drug–drug interaction between tafenoquine and artemisinin combination therapies (ACTs) or the lack of necessary synergism with chloroquine [8] and led to a change in drug label for tafenoquine, limiting its use for countries which have adopted ACT for the first-line treatment of patients with either *P. falciparum* or *P. vivax* malaria. However, alternative explanations for the low efficacy of tafenoquine in the INSPECTOR study have been raised, including suboptimal dosing, particularly in an area with high relapse frequency and high hypnozoite load on the Island of Papua [9, 10].

The 7-day course of primaquine, administered at a high daily dose (1 mg/kg/day), has been proposed as a short-course regimen to improve patient adherence and was found to be non-inferior to the same total dose administered over 14 days (0.5 mg/kg/day) [11]. However, until recently the effectiveness of the 7-day regimen compared to that of the 14-day regimen or a single dose tafenoquine was unknown. The SCOPE study (NCT05879224) was designed as a large multicentre feasibility study conducted in Indonesia and PNG, to determine the safety and feasibility of G6PD testing followed by treatment with the 7-day high dose primaquine regimen for G6PD normal patients, or the 14-day regimen if patients had intermediate G6PD activity and 8-weekly primaquine if patients were G6PD deficient [12]. In parallel to the SCOPE study, the EFFORT trial (NCT04411836) was conducted to evaluate the safety and effectiveness of unsupervised high-dose primaquine (7 mg/kg total dose over 7 days) and single-dose tafenoquine (300 mg) compared to the standard 14-day low-dose primaquine regimen (3.5 mg/kg total dose). The trial was designed as a pragmatic, multi-country, randomised controlled trial in four countries—Cambodia, Ethiopia, Indonesia, and Pakistan. In Indonesia, tafenoquine and primaquine were co-administered with DHA-piperaquine. In Cambodia, tafenoquine and primaquine were administered with artesunate–pyronaridine, while chloroquine was used in Pakistan and Ethiopia. Detailed findings of the EFFORT trial have been reported elsewhere [13]. In brief, the trial showed that overall, both the 7-day high-dose primaquine and single-dose tafenoquine regimens were well tolerated and led to significantly lower risks of *P. vivax* recurrence compared to the standard 14-day low-dose primaquine regimen. The country-specific subgroup analysis for Cambodia and Indonesia challenges the assumption that tafenoquine does not prevent relapses when combined with an ACT. The findings suggest that potential drug-drug interactions may not contribute equally to all antimalarial combinations and raise the possibility that tafenoquine may retain efficacy when combined with an ACT.

## Meeting description

Organised by the Menzies School of Health Research in collaboration with the Papua Community Health and Development Foundation (Yayasan Pengembangan Kesehatan dan Masyarakat Papua, YPKMP) and Tridarma Healthcare Empowerment Foundation (Yayasan Penguatan Kesehatan Masyarakat Tridarma, YPKMT) the dissemination of EFFORT study results in Indonesia took place on May 27, 2025. During the meeting, the final results of the EFFORT study [13] as well as second-quarter observations from, the SCOPE study [12] were presented.

Participants included representatives from the Ministry of Health, the National Agency of Drug and Food Control (Badan Pengawas Obat dan Makanan, BPOM), WHO, Mimika and Batu Bara District Health Offices, Lampung and North Sumatera Provincial Health Offices, members of the Malaria Technical Working Group as well as research teams from Australia, North Sumatera, South Sumatera, and Papua. In total 33 participants attended.

Following the presentation of study outcomes, participants engaged in a preliminary discussion on the potential rollout of different radical cure treatments in Indonesia. This included consideration of key operational and strategic factors such as costing and economic analysis, additional evidence required and planning for reviews by the National Malaria Technical Working Group.

Throughout the meeting, notes were taken and collated to present key discussion points. Discussion points are presented under the following themes in this report: (i) tafenoquine and ACTs: evidence gaps and next steps in Indonesia, (ii) high-dose primaquine as a feasible near-term option, (iii) the need for subnational policies, and (iv) strong political commitment to elimination.

## Tafenoquine and ACTs: evidence gaps and next steps in Indonesia

Stakeholders discussed the potential role of tafenoquine in Indonesia’s malaria elimination strategy and the specific challenges posed by its co-administration with ACTs, particularly the use of DHA–piperaquine, the first-line treatment for patients with uncomplicated malaria due to any species. Tafenoquine offers the significant advantage of single-dose administration that avoids issues of poor adherence, but its current label restricts use to co-administration with chloroquine.

In Cambodia, where tafenoquine was combined with artesunate–pyronaridine, the risk of recurrence at six months was approximately 15% [13]—well below the estimated 80% background risk in the absence of radical cure [14]—suggesting strong anti-relapse efficacy and arguing against a clinically significant drug–drug interaction between tafenoquine and this ACT.

In contrast, results from Sumatera, Indonesia, where DHA–piperaquine was used, were more difficult to interpret [13]. The risk of relapse without radical curative treatment varies considerably in Indonesia. In the eastern province of Papua up to 80% of patients have recurrent *P. vivax* malaria within 6 months [8], whereas in the western province of Sumatera the risk of relapse is significantly lower – about 25% at 6 months [11, 15]. In the EFFORT trial the risk of recurrence at 6 months in patients treated with DHA–piperaquine plus tafenoquine was only 25% [13], but this could simply reflect a low background risk of recurrence rather than effective anti-relapse treatment. However, recurrences in the tafenoquine arm tended to occur later (after four months), and most were genetically heterologous, suggesting reinfection rather than relapse [16] and potentially good efficacy. The number of patients followed beyond 4 months was small, with wide confidence interval, and almost all of the recurrences occurred in the tafenoquine arm, raising concerns of suboptimal efficacy that warrant further investigation [13] (Table 1).

**Table 1:** Evidence gaps identified during meeting mapped to ongoing trials

Evidence gaps identified during meeting	Ongoing trials that address the evidence gap
Efficacy and safety of 300 mg tafenoquine with different ACTs	
Efficacy of 300 mg tafenoquine with DHA-Piperaquine	Tafenoquine and ACTs (TADORE- Plus) study (NCT07060794) A Revised Tafenoquine Dose to Improve Radical Cure for Vivax Malaria (TADORE) study (NCT06148792) Tafenoquine Combinations for Improved Radical Cure Efficacy of Plasmodium Vivax study (NCT07533136)
Efficacy of 300 mg tafenoquine with Artemether-lumefantrine	Southeast Asia Dose Optimization of Tafenoquine (SEADOT) study (NCT04704999) A Revised Tafenoquine Dose to Improve Radical Cure for Vivax Malaria (TADORE) study (NCT06148792) Tafenoquine Combinations for Improved Radical Cure Efficacy of Plasmodium Vivax study (NCT07533136)
Efficacy of 300 mg tafenoquine with artesunate–pyronaridine	Tafenoquine and ACTs (TADORE- Plus) study (NCT07060794)
Efficacy and safety of different tafenoquine dosing (with and without ACTs)	
Efficacy of higher weight-based dosing	A Revised Tafenoquine Dose to Improve Radical Cure for Vivax Malaria (TADORE) study (NCT06148792)
Efficacy of higher fixed doses	Southeast Asia Dose Optimization of Tafenoquine (SEADOT) study (NCT04704999) ACT vs CQ With Tafenoquine for *P. vivax* Mono-infection (ACTQ) study (NCT05788094)

In light of these findings meeting participants emphasised the importance of conducting additional prospective clinical trials that include a control arm receiving delayed radical cure, to enable clearer interpretation of tafenoquine efficacy when co-administered with DHA–piperaquine and other ACTs in areas of low background risk. Such studies are now underway, including trials in Indonesia. These are expected to provide more definitive evidence on the efficacy of 300 mg tafenoquine with DHA-piperaquine (Table 1) .

Stakeholders also discussed whether the currently approved tafenoquine dose (300 mg) is sufficient in all settings, even when co-administered with chloroquine. Participants discussed the evidence that increasing the tafenoquine dose could help overcome a potential reduction in antirelapse efficacy due to any pharmacological interactions or context-specific factors such as high parasite burden or early relapse patterns [9]. A series of studies is currently underway in multiple countries including Indonesia to explore the safety and efficacy of higher fixed or weight-based tafenoquine dosing strategies (Table 1).

## High-dose primaquine as a feasible near-term option

Meeting attendees discussed the role of high-dose primaquine as an immediately actionable option for improving radical cure of *P. vivax* malaria in Indonesia. The previous IMPROV trial included study sites in North and South Sumatera, and highlighted that in G6PD normal patients, the high-dose short-course regimen (1 mg/kg/day for 7 days) was well tolerated and highly effective [11]. In contrast to tafenoquine, high-dose primaquine in combination with an ACT is already recommended by WHO [7] and was shown in the EFFORT trial to be both effective and well tolerated, with no recurrences reported within six months of follow-up at the Indonesia study site [13]. In view of these findings, high-dose primaquine was considered a viable near-term alternative to the currently used 14-day low-dose regimen.

Participants noted, however, that the widespread adoption of high-dose primaquine will require scaled-up access to G6PD testing to ensure patient safety. Quantitative point-of-care G6PD diagnostics, such as the STANDARD G6PD, are available in Indonesia, but integration into policy and routine clinical workflows has not yet occurred. Stakeholders emphasised the importance of strengthening G6PD testing infrastructure and training to support safe delivery of higher-dose regimens.

The need for ongoing implementation research, including the SCOPE study [12], was highlighted as an important step towards evaluating the feasibility of high-dose primaquine under programmatic conditions. The findings of this study are expected to inform practical guidance on how high-dose regimens could be introduced at scale, including in remote and decentralized settings.

## The need for subnational policies

Stakeholders highlighted the importance of tailoring malaria elimination strategies to Indonesia’s highly heterogeneous transmission settings. The risk of *P. vivax* relapse, local transmission intensity, and operational capacity vary substantially across the archipelago, and this has major implications for both the choice of radical cure regimen and how it should be implemented. For example, in Papua, where relapse risk is high and early relapses are common, more aggressive or intensified radical cure strategies with higher doses may be warranted.

Participants noted that a uniform national policy may not sufficiently address these diverse realities and that subnational policies—guided by local data and operational feasibility—could play a critical role in improving programmatic outcomes. Subnational policies should include tailoring the choice of drug regimen (e.g., high-dose primaquine vs tafenoquine) and also decisions regarding G6PD testing strategies, supervision requirements, and prioritisation of resources. Aligning treatment policies with local epidemiology was seen as essential for maximising the impact of radical cure interventions while ensuring safety and efficiency.

## Strong political commitment to elimination

Stakeholders highlighted Indonesia's strong political momentum behind its malaria elimination goals, that was reinforced at the 9th Asia Pacific Leaders’ Summit on Malaria Elimination held in Bali in June 2025. Participants at the meeting stressed that this unprecedented political momentum must be matched by resources, multisectoral coordination, and robust implementation—including support for expanded radical cure strategies—to translate commitments into real-world progress across Indonesia’s diverse epidemiological zones.

## Conclusion

The stakeholder meeting highlighted both the promise of new radical cure options and the key evidence gaps that remain for Indonesia. Meeting attendees emphasised the need for further studies of tafenoquine with DHA–piperaquine and other ACTs, while recognising high-dose primaquine as an immediately implementable option pending strengthened G6PD testing capacity. The discussion underscored the importance of tailoring strategies to Indonesia’s diverse transmission settings, supported by strong national commitment to malaria elimination.

## Data Availability

No datasets were generated or analysed during the current study.

## Abbreviations

ACT: Artemisinin combination therapies
BPOM: Badan Pengawas Obat dan Makanan
DHA-piperaquine: Dihydroartemisinin-piperaquine
G6PD: Glucose-6-phosphate dehydrogenase
WHO: World Health Organization
YPKMP: Yayasan Pengembangan Kesehatan dan Masyarakat Papua
YPKMT: Yayasan Penguatan Kesehatan Masyarakat Tridarma
